# Association of Variability in the DDAH1, DDAH2, AGXT2 and PRMT1 Genes with Circulating ADMA Concentration in Human Whole Blood

**DOI:** 10.3390/jcm11040941

**Published:** 2022-02-11

**Authors:** Juliane Hannemann, Julia Zummack, Jonas Hillig, Leonard Rendant-Gantzberg, Rainer Böger

**Affiliations:** Institute of Clinical Pharmacology and Toxicology, University Medical Center Hamburg-Eppendorf, 20246 Hamburg, Germany; julia.zummack@web.de (J.Z.); jonas.hillig@alice-dsl.net (J.H.); leonard.rendant-gantzberg@bts-ev.de (L.R.-G.); boeger@uke.de (R.B.)

**Keywords:** genetic variability, genotyping, nitric oxide

## Abstract

Asymmetric dimethylarginine is an endogenous inhibitor of nitric oxide synthesis and a cardiovascular risk factor. Its regulation has been studied extensively in experimental models, but less in humans. We studied common single-nucleotide polymorphisms (SNPs) in genes encoding for enzymes involved in ADMA biosynthesis and metabolism, i.e., PRMT1, DDAH1, DDAH2, and AGXT2, and assessed their associations with blood ADMA concentration in 377 unselected humans. The minor allele of DDAH1 SNP rs233112 was significantly more frequent in individuals with ADMA in the highest tertile or in the highest quartile, as was the major allele of DDAH2 rs805304. A combined genotype comprising both SNPs showed a significant genotype–phenotype association, with increasing ADMA concentration by an increasing number of inactive alleles. SNPs in the AGXT2 and PRMT1 genes showed no significant associations with blood ADMA concentration. Our study provides comprehensive evidence that DDAH1 and DDAH2 are the major enzymes regulating blood ADMA concentration, whilst PRMT1 indirectly affects ADMA, and AGXT2 may act as a back-up enzyme in ADMA metabolism under pathophysiological conditions only.

## 1. Introduction

Asymmetric dimethylarginine (ADMA) is an endogenous inhibitor of nitric oxide (NO) synthesis. Both in vitro and in vivo, ADMA concentration-dependently inhibits the conversion of ^15^N-L-arginine into ^15^NO_3_^−^ [1,2]; administration of L-arginine in vivo overcomes the inhibitory effects of ADMA and restores endothelium-dependent vasodilation [3]. Therefore, ADMA has been recognized as an important regulator of NO availability; its circulating concentration is associated with total mortality and cardiovascular disease (CVD) events [4].

**Figure 1:**
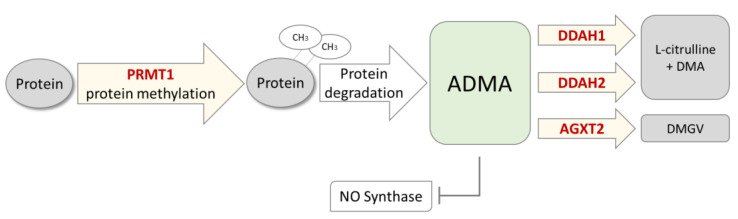
Scheme of enzymatic pathways for biosynthesis and metabolism of asymmetric dimethylarginine (ADMA). L-arginine residues within specific proteins are subject to methylation by protein arginine N-methyltransferases (PRMTs), amongst which PRMT1 has been identified to be the major isoform generating ADMA. Free ADMA is released during physiological protein turnover. ADMA is a competitive inhibitor of nitric oxide (NO) synthase. ADMA is mainly degraded by dimethylarginine dimethylaminohydrolases (DDAH1 and DDAH2) into L-citrulline and dimethylamine (DMA). It may be cleaved by an alternative pathway through alanine glyoxylate aminotransferase 2 (AGXT2), resulting in the formation of asymmetric dimethylguanidinovaleric acid (DMGV). Enzymes of which we have analyzed genetic variance in this study are marked in bold red type within the yellow arrows.

ADMA is formed during posttranslational protein modification by protein arginine N-methyltransferases (PRMTs) that introduce up to two methyl groups into L-arginine residues of specific proteins (for review, cf. [5]). Among nine PRMT isoforms that have been identified thus far, PRMT1 appears to be the isoform responsible for the majority of asymmetric protein demethylation, and, thus, ADMA production [6]. Free ADMA is released during cellular turnover of methylated proteins. Its concentration in plasma and tissues is controlled by either of two isoforms of dimethylarginine dimethylaminohydrolase (DDAH) [7], of which DDAH1 appears to be the major enzyme that is highly expressed in kidneys and liver and regulates plasma ADMA concentration in animal models [8], whilst DDAH2 can be found in vascular tissues and lungs [7], where it is involved in the regulation of NO-mediated vasodilation [8]. Alanine glyoxylate aminotransferase-2 (AGXT2) is a mitochondrial enzyme that is able to utilize ADMA and its congener, symmetric dimethylarginine (SDMA) as alternative substrates [9]; however, the physiological role of AGXT2 in controlling ADMA concentration has not yet been fully understood, as the majority of experiments that showed its involvement in ADMA metabolism were performed using overexpression models [10]. In a human genome-wide association study, we did not find an association of the AGXT2 gene with ADMA [11]. Figure 1 depicts the major pathways of ADMA biosynthesis and metabolic degradation, highlighting the genes that were analyzed in this study.

To further clarify the physiological roles of the different enzymes involved in ADMA biosynthesis and degradation in humans, we analyzed genetic polymorphisms in each of the corresponding genes that might be linked to altered dimethylarginine levels, nitric oxide generation, or vascular disease. We identified nine single nucleotide polymorphisms (SNPs) in the PRMT1, DDAH1, DDAH2, and AGXT2 genes that fulfilled this criterion and for which the expected minor allele frequencies were sufficiently high to allow for genotype–phenotype analyses in a medium-sized cohort. Thus, the aim of the present study was to analyze common single nucleotide polymorphisms in four genes relating to ADMA and to assess the associations of the major and minor alleles of these genes with circulating ADMA concentration in a randomly chosen human cohort.

## 2. Materials and Methods

### 2.1. Study Participants and Protocol

We included 377 adult human subjects who had consented to have their ADMA and L-arginine levels measured in dried blood spots for a variety of motivations, ranging from an individual history of cardiovascular disease to the presence of cardiovascular risk factors, as well as to a primary prevention approach to biomarker analysis. No specific inclusion and exclusion criteria were applied to define this unselected study cohort. Each individual had filled a short, structured questionnaire asking for medical history; presence of cardiovascular risk factors (i.e., hypertension, hypercholesterolemia, smoking, and diabetes); and additional variables such as systolic and diastolic blood pressure, total cholesterol, LDL and HDL cholesterol, triglyceride, and creatinine serum levels if known to the individual. Each individual had six blood spots drawn from one fingertip; three blood spots were used for ADMA analysis and three blood spots were used for isolation of genomic DNA. 

The samples and questionnaires were anonymized after the participants had received their individual reports on biomarker levels, and all genomic analyses were performed with strictly anonymized data in agreement with current data protection regulations after the anonymization code had been discarded.

### 2.2. Assessment of ADMA Concentration from Dried Blood Spots

We have previously published the method of analysis of ADMA from dried blood spots in detail [12]. Briefly, three dried blood spots from each subject were collected in reaction tubes and incubated with 300 µL of elution buffer on an orbital shaker for 20 min. Samples were centrifuged at 3000× *g*, and two aliquots of 75 µL each were used for analysis after acylation (60 min, room temperature). Analysis of ADMA was performed using commercially available ELISA assays (DLD Diagnostika, Hamburg, Germany) according to the manufacturer’s instructions. The ADMA ELISA had previously been validated by our group [13].

### 2.3. DNA Isolation from Dried Blood Spots

DNA was isolated from dried blood spots using a salting-out procedure modified from Shaik et al. [14]. Briefly, three punches of dried blood (6 mm each) were collected in a 2 mL reaction tube and incubated in 750 µL methanol by gently shaking for 15 min at room temperature. The punches were allowed to air dry for 15 to 30 min and were then transferred to a clean 2 mL reaction tube. A total of 500 µL of lysis buffer (20 mM EDTA, pH 8.0; 30 mM Tris-HCl; 5 mM MgCl_2_; 1% Triton X-100 (*v*/*v*); 3% SDS (*w*/*v*)) were added and allowed to incubate for 30 min at 80 °C and 400 rpm on an Eppendorf Thermomixer. Subsequently, 100 µg of proteinase K was added, followed by a 60 min incubation at 60 °C and 400 rpm. A total of 200 µL of 6 M NaOH was added, and the sample was centrifuged for 15 min at room temperature at 3000× *g*. The clear upper phase was transferred into a clean 1.5 mL reaction tube, and DNA was precipitated by 3 M sodium acetate (pH 5.0) and absolute ethanol overnight at −20 °C. Pellets were washed once with 70% ethanol, resuspended in 20 µL H_2_O, and dissolved over night at 4 °C and 400 rpm on a thermomixer. DNA concentration was determined using a nanophotometer NP60 (Implen, Munich, Germany).

### 2.4. Genotyping

We genotyped three SNPs in the DDAH1 gene (rs1241321, rs480414, and rs233112), two SNPs in the DDAH2 gene (rs805304 and rs2272592), two SNPs in the AGXT2 gene (rs16899974 and rs37369), and two SNPs in the PRMT1 gene (rs1041588 and rs975484). These SNPs were selected on the basis of a literature search for SNPs that have been reported to be associated with differences in ADMA concentration, differences in nitric oxide-related vascular function, or cardiovascular diseases (Appendix A). Details of published information on the selected SNPs is given in Appendix A Table A1. SNP genotyping was performed using single-tube human TaqMan SNP Genotyping Assays (Thermofisher, Waltham, MA, USA). Each reaction mix contained 10 ng template DNA, 0.5 µL specific TaqMan SNP Genotyping Assay, and 5 µL of 2xTaqPath ProAmp™ Mastermix in a total reaction volume of 10 µL. The PCR was performed in a QuantStudio™ 5 Real-Time PCR System (Thermofisher, Waltham, MA, USA) using the following PCR program: pre-read of 30 s at 60 °C, enzyme activation at 95 °C for 5 min, 40 cycles of denaturation (5 s, 95 °C) and annealing (30 s, 60 °C), followed by a last post-read of 30 s at 60 °C. Allelic calls were identified by Quant Studio Design and Analysis Software (Thermofisher, Waltham, MA, USA).

### 2.5. Statistical Analyses

All statistical analyses were performed using SPSS (version 21; IBM Corporation, Armonk, NY, USA) and GraphPad Prism (version 6.01, GraphPad Software, San Diego, CA, USA). All variables were tested for normal distribution using the Kolmogorov–Smirnov test. Differences between groups were tested for significance by using either the nonparametric Mann–Whitney *U* test for two groups or the Kruskal–Wallis analysis of variance for more than two groups. Allele frequencies of the genes of interest were compared to the European reference population or between groups using contingency tables and Fisher’s exact test. Differences in ADMA concentrations between genotypes were tested for statistical significances by ANOVA followed by the Scheffé f-test, and allele frequencies between ADMA tertiles and quartiles were tested for significance by using the χ^2^ test. Data are presented as median with interquartile range, or as mean with standard deviation. For all tests, *p* < 0.05 was considered significant.

## 3. Results

### 3.1. Baseline Characteristics of the Study Participants

We analyzed 377 human subjects with a mean age of 59.4 ± 13.1 years. Both sexes were evenly distributed (50.9% males); the prevalence of CVD and risk factors is given in Table 1. 

### 3.2. Distribution of ADMA Concentration in the Study Cohort

**Figure 2:**
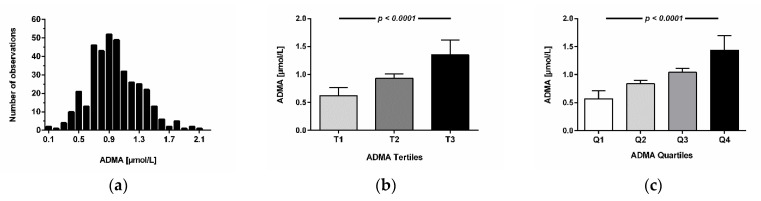
(**a**) Histogram depicting the distribution of circulating ADMA concentrations in the study cohort. Data are shown as absolute frequencies of ADMA concentrations in 377 adult humans split up in 0.1 µmol/L intervals. (**b**) ADMA concentration in tertiles of ADMA (first tertile, N = 121; second tertile, N = 128; third tertile, N = 128). (**c**) ADMA concentration in quartiles of ADMA (first quartile, N = 88; second quartile, N = 99; third quartile, N = 96; fourth quartile, N = 94). Data are depicted as mean ± SD. *p*-values denote statistical significance for two-sided one-way ANOVA.

Mean circulating ADMA concentration in the study cohort was 0.98 ± 0.35 µmol/L. The distribution of ADMA concentration in the study cohort is shown in as a histogram in Figure 2a. Individuals with high ADMA had a significantly higher mean age than individuals with low ADMA; the proportion of males significantly increased with increasing tertiles of ADMA (Table 1).

We further analyzed the association of circulating ADMA with CVD and CVD risk factors. We found no significant trends for higher prevalence of hypertension, diabetes mellitus, history of myocardial infarction, or smoking status with increasing tertiles of ADMA (Table 1). There was a trend towards fewer patients with diabetes mellitus with higher ADMA tertiles; however, due to the relatively small number of patients with diabetes, this did not reach statistical significance. We neither observed significant trends for systolic nor for diastolic blood pressure, cholesterol, and triglyceride levels across ADMA tertiles. However, individuals in the highest ADMA tertile had significantly higher serum creatinine concentrations than those in the lowest ADMA tertile (*p* = 0.038). Mean ADMA concentrations in tertiles and in quartiles of ADMA are depicted in Figure 2b,c.

### 3.3. Allele Frequencies of the SNPs in the Study Cohort Compared to the European Reference Population

The prevalence of the major allele of PRMT1 rs10415880 was significantly higher, and that of DDAH1 rs2331112 was slightly but significantly lower than those expected on the basis of allele frequencies reported for the European reference population of the 1000 Genomes project [15]. For all other SNPs investigated in this study, the frequencies of the major and minor alleles did not deviate significantly from those reported for the 1000 Genomes project (Table 2). 

### 3.4. Association of SNPs with ADMA Concentration

We observed a significant association of DDAH1 rs233112 genotypes with mean ADMA concentration (*p* = 0.027 for trend across genotypes); individuals homozygous for the minor allele had a significantly higher mean ADMA concentration than in carriers homozygous for the major allele. For all other SNPs, there were no significant associations of ADMA concentration with genotypes. Table 3 summarizes the associations of all SNPs with ADMA concentration.

**Figure 3:**
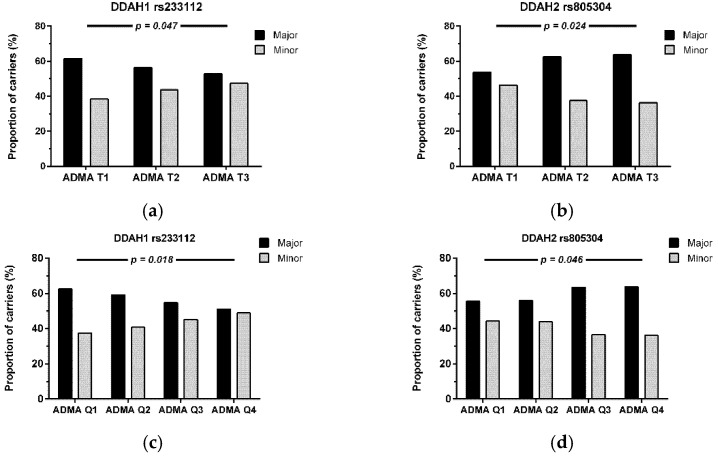
Allele frequencies for the major and minor allele of DDAH1 SNP rs233112 (**a**,**c**) and DDAH2 SNP rs805304 (**b**,**d**) separated by tertiles (**a**,**b**) and quartiles of ADMA (**b**,**d**), respectively. *p*-values denote statistical significance in χ^2^ test for trend.

We next subdivided individuals by tertiles and quartiles of ADMA concentration and assessed differences in allele frequencies for all SNPs across ADMA tertiles and quartiles. With increasing tertiles of ADMA, the frequency of the major allele of DDAH1 rs233112 decreased significantly (*p* = 0.047; Figure 3a); by contrast, the frequency of the major allele of DDAH2 rs805304 increased significantly (*p* = 0.024; Figure 3b). When we analyzed allele frequencies for quartiles of ADMA, the differences in allele frequencies of DDAH1 rs2331112 and DDAH2 rs805304 retained statistical significance (*p* = 0.018 and *p* = 0.046, respectively; Figure 3c,d).

**Figure 4:**
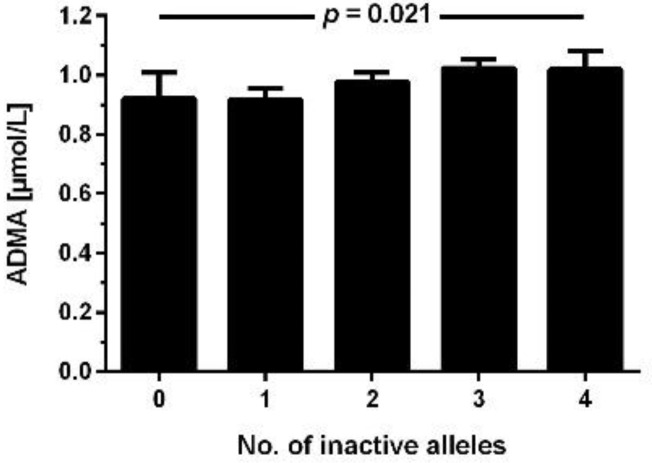
ADMA concentration according to the number of inactive alleles in the combined genotype of DDAH1 rs2331112 and DDAH2 rs805304. Data are mean ± S.E.M. *p* denotes statistical significance in linear regression analysis.

We then constructed a combined genotype of the DDAH1 rs233112 and DDAH2 rs805304 polymorphisms (inactive alleles: minor allele for rs233112, major allele for rs805304) and analyzed its association with ADMA concentration. In this analysis, “zero inactive alleles” refers to individuals homozygous for the major allele of rs2331112 and the minor allele of rs805304; “4 inactive alleles” refers to individuals homozygous for the minor allele of rs233112 and the major allele of rs805304. Individuals with 1–3 inactive alleles were heterozygous for one or both SNPs. Figure 4 demonstrates that ADMA concentration significantly increased with an increasing number of inactive alleles for the combined genotype (*p* = 0.021 for trend). 

## 4. Discussion

This study has two major results. Firstly, in an unselected and diverse cohort of human subjects, DDAH1 and DDAH2 were the two genes that were significantly related with circulating ADMA concentration in whole blood. Secondly, neither AGXT2 nor PRMT1 showed significant associations with ADMA concentration in whole blood.

This study is the first to comprehensively investigate SNPs in all four known genes encoding for enzymes involved in dimethylarginine biosynthesis or metabolism, and that have previously been reported to be associated with high ADMA and/or NO-dependent vascular function, with blood ADMA levels in a relatively large human cohort. Whilst our first finding confirms previous reports from genome-wide association studies [11] and observational and experimental studies [16,17,18] that DDAH isoenzymes are the major enzymes controlling circulating ADMA concentration in humans, the second finding is novel, as there have been no prior studies of similar size that analyzed the associations of AGXT2 and PRMT with circulating ADMA concentration in humans. Nonetheless, there has been debate about the differential roles of the enzymes involved in the biosynthesis and degradation of ADMA in regulating ADMA concentration. This was mainly fostered by experimental results that showed the influence of overexpressing or deleting the genes encoding for one of the respective enzymes.

Achan and co-workers [19] estimated that about 80% of the total ADMA synthesized in humans is metabolically cleaved by DDAH(s). Genetic knockdown studies of either DDAH1 or DDAH2 suggested that DDAH1 is the major DDAH isoform involved in regulating circulating ADMA concentration [8]. Plasma ADMA concentration increased by 30% (heterozygous DDAH knockout; [18]) and 120% (homozygous DDAH1 knockout; [20]) in two independent DDAH1 knockout mouse models. These knockout mouse models therefore confirmed the primary role of DDAH1 in ADMA metabolism. By contrast, there was no change in plasma ADMA in homozygous DDAH2 knockout mice [17], whereas tissue concentrations of ADMA in kidneys and myocardium were 93% and 33% higher than in wild-type littermates, respectively. Taken together, these data suggest that ADMA concentration circulating in blood is regulated primarily by DDAH1 and independently from ADMA tissue concentrations, at least in heart and kidneys. For AGXT2, one study demonstrated that homozygous knockout of this gene in mice increased plasma ADMA concentration by some 25% [21]. The same authors estimated that about 20% of variability in (log)ADMA and (log)SDMA concentrations in humans is caused by AGXT2. 

Overexpression of human DDAH1 in mice resulted in significantly lower circulating and tissue ADMA concentrations [16], whilst DDAH1 knockout mice showed significantly elevated ADMA plasma concentration [18]. By contrast, DDAH2 knockout mice did not show elevated ADMA plasma concentration despite altered ADMA tissue concentration in myocardium and kidneys and impaired endothelium-dependent vascular reactivity [17]. 

In our study we found that the minor allele of DDAH1 rs233112 was significantly associated with high ADMA blood levels. Previous investigators had reported that the minor allele of this SNP was associated with arterial stiffness and increased pulse wave reflection [22]. High ADMA, by inhibiting NO generation, may cause arterial stiffness and increase arterial pulse wave reflections [23], thus offering a mechanistic explanation for this genotype–clinical phenotype association. However, genetic variation in DDAH1 was found to be related to circulating ADMA concentration but not to endothelium-dependent vasodilation in a large, population-based study [24], nor were any DDAH2 SNPs associated with vascular reactivity in this study. Therefore, we must be careful in translating experimental findings from genetically engineered rodent models into human biology, and DDAH genotype–phenotype relationships in humans clearly deserve further careful investigation.

The second SNP that was significantly associated with blood ADMA levels was DDAH2 rs805304, of which the major allele was associated with higher ADMA. The DDAH2 gene and, more specifically, this SNP, appear to have a complex relationship with ADMA metabolism. In previous studies, this promoter polymorphism was associated with the prevalence of arterial hypertension [25] and with ADMA levels in type 2 diabetes subjects with renal function impairment [26], whilst none of these associations was found in another study [27]. In a study comprising 473 patients with acute myocardial infarction and 447 controls, the minor allele of DDAH2 rs805304 was significantly associated with a decreased risk of myocardial infarction [28]. Another DDAH2 SNP (rs805305) turned out to be protective in septic shock by regulating ADMA concentration through DDAH activity [29]. Since both SNPs, rs805304 and rs805305, are in strong linkage disequilibrium (R^2^ = 1.0), our findings for rs805304 in the present study are assumed to be equally valid for rs805305. In line with these prior observations, we recently gathered evidence that carriers of the major allele of DDAH2 rs805304 had a greater increase in ADMA during hypoxia [30]. Taken together, these previous findings and our present data may suggest that DDAH2 underlies transcriptional regulation in pathophysiological conditions (myocardial infarction, diabetes, hypoxia, sepsis), and genetic variability in this gene may impair this regulatory role. Future studies should aim to further clarify the pathophysiological role of DDAH2 versus DDAH1 in humans.

Data showing that AGXT2 metabolizes ADMA came mostly from models in which the AGXT2 gene was overexpressed under the control of promoters that ensured high expression levels of the gene product [10,31]. Another mouse study showed that infusion of beta-aminoisobutyrate (BAIB), a major substrate of AGXT2, may displace ADMA from this enzyme in mice and cause a moderate increase in ADMA concentration [32]. Nonetheless, it has remained unclear as to whether AGXT2 contributes to the physiological control of ADMA concentration in humans. Kittel and co-workers reported that the two coding AGXT2 SNPs rs27269 and rs16899974 were associated with BAIB and SDMA, but not with ADMA plasma concentration in healthy humans [33]. This was strongly supported by our finding from a genome-wide association study that AGXT2 only showed genome-wide significant association with SDMA but not with ADMA concentration [11]. Our present findings further support the notion that, although AGXT2 is catalytically able to utilize ADMA as a substrate, its physiological role in humans as an ADMA-degrading enzyme may be limited. The main catalytic activity of AGXT2 is directed towards the conversion of alanine and glyoxylate to pyruvate and glycine [9]; both ADMA and SDMA may act as alternative substrates for this enzyme. In conditions of impaired DDAH expression or activity, AGXT2 may step in and help to maintain low dimethylarginine concentrations. In line with this hypothesis, we have recently observed that exposure of healthy human subjects to six months of chronic intermittent hypoxia leads to a gradual elevation of plasma ADMA, whilst simultaneously, plasma SDMA gradually decreased [34]. As AGXT2 degrades both ADMA and SDMA, whilst DDAH is inactive towards SDMA, this pattern of metabolite changes in plasma would be consistent with downregulation of DDAH in hypoxia, as has been shown experimentally, and compensatory upregulation of AGXT2. In a recent study in 750 elderly Japanese individuals, a loss-of-function haplotype comprising four SNPs in AGXT2 was positively associated with blood pressure and serum glucose levels; individuals homozygous for this haplotype had significantly elevated ADMA levels [35]. Thus, the physiological and pathophysiological roles of AGXT2 in dimethylarginine metabolism in humans require further study.

PRMT1 has been described to be the major protein arginine methyltransferase that is responsible for asymmetric demethylation of proteins, accounting for 70–80% of ADMA generation [36]. However, there are at least five additional PRMTs that asymmetrically dimethylate proteins—i.e., PRMT2, PRMT3, PRMT4 (CARM1), PRMT6, and PRMT8; they are categorized as type 1 methyltransferases [37]. Dimethylation of arginine residues within specific proteins results in generation of free ADMA only after hydrolytic degradation of the respective proteins and outbound transport of ADMA from the cytoplasm via the y^+^ amino acid transporter system [38]. Thus, PRMTs rather indirectly affect blood ADMA concentration, a fact that may explain why in our study there was no significant association of PRMT1 SNPs with blood ADMA concentration.

As we did not actually measure standard laboratory parameters but used those reported by the study participants, our correlation analyses of ADMA with standard laboratory parameters do not support major conclusions. It is interesting to note, however, that the previously described correlation of ADMA with renal function was reproduced in this study by showing a significant association of ADMA tertiles with serum creatinine concentration [39,40]. DDAH1 is highly expressed in renal tissue, as is AGXT2; the correlation of ADMA with creatinine may therefore point to dysregulation of the expression and/or activity of these ADMA-metabolizing enzymes in the kidneys rather than to reduced excretion of unchanged ADMA into the urine [41,42]. Elevated ADMA concentration in kidney disease may thus explain the high incidence of cardiovascular co-morbidity in this patient population. 

Our study has a number of strengths and limitations. We analyzed whole blood concentration of ADMA on the basis of a previously described and validated dried blood spot assay [12]. Whole blood concentration of ADMA was shown to be higher than its plasma concentration when intra-individual comparisons were made (0.98 ± 0.28 µmol/L vs. 0.78 ± 0.05 µmol/L; [12]). Nonetheless, we demonstrated a close correlation between ADMA concentration in dried whole blood spots and in plasma [12]. Whilst limitation of sample size is always a drawback, one strength of our study results from the fact that we included an unselected, diverse cohort, and that we did not influence the association of genotypes and phenotype by any active intervention nor by selection of a specific patient population. This is the first study to analyze genetic variability in the four major genes linked to biosynthesis and metabolism of ADMA. Nonetheless, we were unable to quantitatively estimate the relative contributions of DDAH1, DDAH2, and AGXT2 to ADMA metabolism, as we did not measure DDAH and AGXT2 activities in this study.

## 5. Conclusions

In conclusion, our study provides comprehensive evidence that amongst all enzymes involved in dimethylarginine synthesis and degradation, DDAH1 and DDAH2 are the ones that most directly influence blood ADMA concentration in humans.

## Figures and Tables

**Table 1:** Baseline characteristics of the study participants.

	All	Low ADMA (First Tertile)	Medium ADMA (Second Tertile)	High ADMA (Third Tertile)
Demographics	N = 377	N = 121	N = 128	N = 128
Age, years	59.4 ± 13.1	58.5 ± 13.4	57.0 ± 12.9	62.6 ± 12.6 *
Age range, years	18–92	22–91	21–92	18–90
Male sex	192 (50.9)	47 (38.8)	65 (50.8) ^#^	80 (62.5) ^#^
Female sex	185 (49.1)	74 (61.2)	63 (49.2) ^#^	48 (38.5) ^#^
ADMA concentration in whole blood				
ADMA, µmol/L	0.98 ± 0.35	0.62 ± 0.15	0.93 ± 0.07 *	1.35 ± 0.27 *
Laboratory parameters				
Systolic BP, mm Hg	128.9 ± 16.5	129.9 ± 15.9	130.3 ± 17.7	126.8 ± 15.7
Diastolic BP, mm Hg	77.9 ± 9.5	77.2 ± 10.2	79.2 ± 9.0	77.2 ± 9.3
Total cholesterol, mmol/L	5.08 ± 1.33	5.30 ± 1.00	4.82 ± 1.50	5.09 ± 1.45
Triglycerides, mmol/L	1.45 ± 1.30	1.62 ± 1.38	1.51 ± 1.09	1.23 ± 1.45
Creatinine, mmol/L	0.91 ± 0.24	0.80 ± 0.22	0.93 ± 0.19	1.00 ± 0.29 *
CV risk factors				
History of MI	39 (10.3)	14 (11.6)	7 (5.5)	18 (14.1)
Family history of heart disease	88 (23.3)	32 (26.4)	27 (21.1)	29 (22.7)
Diabetes mellitus	35 (9.3)	18 (14.9)	8 (6.3)	9 (7.0)
Hypertension	53 (14.1)	18 (14.9)	14 (10.9)	21 (16.4)
Congestive heart failure	3 (0.8)	1 (0.8)	1 (0.8)	1 (0.8)
Active smoker	43 (11.4)	14 (11.6)	13 (10.2)	16 (12.5)

Abbreviations: MI, myocardial infarction; ADMA, asymmetric dimethylarginine; CV, cardiovascular. * *p* < 0.05 for significant difference of continuous variables from first tertile of ADMA (univariate ANOVA with Scheffé f-test). ^#^ *p* < 0.05 for significant difference of categorical variables from first tertile of ADMA (Pearson’s χ^2^ test).

**Table 2:** Allele frequencies for single nucleotide polymorphisms in the DDAH1 and DDAH2 genes.

Gene/SNP	Position	Genetic Consequence	Major/Minor Allele	Measured Allele Frequency	Expected Allele Frequency *	*p*
PRMT1						
rs10415880	Intron 8	Intron variant	G/A	0.718/0.282	0.658/0.342	0.008
rs975484	-	Intron variant	C/G	0.730/0.270	0.738/0.262	0.696
DDAH1						
rs1241321	Intron 1	Intron variant	A/G	0.670/0.330	0.712/0.288	0.057
rs233112	Exon 6	3’ UTR variant	T/C	0.568/0.432	0.617/0.383	0.038
rs480414	Intron 1	Intron variant	G/A	0.735/0.265	0.698/0.302	0.092
DDAH2						
rs805304	-	2 kb upstream variant	T/G	0.601/0.399	0.639/0.361	0.102
rs2272592	-	2 kb upstream variant	C/T	0.810/0.190	0.844/0.156	0.064
AGXT2						
rs16899974	Exon 1	Missense variant	C/A	0.753/0.247	0.773/0.227	0.325
rs37369	Exon 13	Missense variant	C/T	0.907/0.093	0.913/0.087	0.640

* Expected allele frequencies were calculated using the allele distributions in the European population of the 1000 Genomes Project (N = 1006) [15]. The *p*-values denote the level of statistical significance for the comparison of absolute frequencies of the minor and major allele in Fisher’s exact test (two-sided). A *p* < 0.05 was considered significant. Abbreviations: AGXT2, alanine glyoxylate aminotransferase-2; DDAH1, dimethylarginine dimethylaminohydrolase-1; DDAH2, dimethylarginine dimethylaminohydrolase-2; PRMT1, protein arginine N-methyltransferase-1.

**Table 3:** Mean ADMA concentration separated by genotypes for each of the SNPs.

	Homozygous Major	Heterozygous	Homozygous Minor	*p* for Trend
PRMT1				
rs10415880	0.96 ± 0.32	1.00 ± 0.35	0.97 ± 0.30	0.642
rs975484	0.98 ± 0.35	1.00 ± 0.35	0.88 ± 0.29	0.275
DDAH1				
rs1241321	1.00 ± 0.38	0.97 ± 0.32	0.90 ± 0.32	0.286
rs233112	0.92 ± 0.39	0.99 ± 0.32	1.03 ± 0.35	0.027
rs480414	0.96 ± 0.38	1.00 ± 0.31	0.94 ± 0.31	0.333
DDAH2				
rs805304	1.01 ± 0.32	0.95 ± 0.34	0.98 ± 0.41	0.157
rs2272592	0.97 ± 0.37	0.98 ± 0.32	1.02 ± 0.25	0.476
AGXT2				
rs16899974	0.97 ± 0.35	0.99 ± 0.33	1.14 ± 0.40	0.232
rs37369	0.99 ± 0.35	0.94 ± 0.31	0.95 ± 0.18	0.156

Data are mean ± SD. *p*-values were calculated by Kruskal–Wallis test for independent samples.

## Data Availability

The data presented in this study are available on request from the corresponding author.

## Appendix A

### Selection and Clinical Significance of the Selected Single-Nucleotide Polymorphisms

In this study, we aimed to investigate whether genetic variance in the genes encoding for enzymes relating to the biosynthesis and metabolism of ADMA are associated with ADMA concentration in human whole blood. The major enzymes involved in these activities are depicted in Figure 1 in the main manuscript. Genetic variability studies on the corresponding genes have been performed previously by several groups of investigators. Table A1 provides an overview of the SNPs that were selected for this study, including references to previous studies linking variability to outcome parameters related to nitric oxide-mediated functions. We made a selection of references relating the SNPs in DDAH1, DDAH2, and AGXT2 to clinical parameters linked to the topic of our present study, whilst there is very little published information available for the PRMT1 gene. Amongst the many known SNPs in each of the genes of interest for our study, many are in linkage disequilibrium (LD) based on information available in the LDLink database of the National Cancer Institute, USA (https://ldlink.nci.nih.gov/; last accessed 8 January 2022). We therefore decided for one tag SNP out of a specific group in order to identify SNPs spread over the whole gene, even if no clinical associations have been published yet.

**Table A1:** Summary of SNPs selected for this study and their previously published associations with clinical parameters.

Gene/SNP	Position	Genetic Consequence	Clinical Rationale	Related References
DDAH1				
rs1241321	Intron 1	Intron variant	Minor allele associated with higher risk of all-cause mortality and the combined endpoint of cardiovascular death, nonfatal myocardial infarction, and stroke.	[43]
rs233112	Exon 6	3’ UTR variant	Minor allele associated with arterial stiffness and pulse wave reflection.	[22]
rs480414	Intron 1	Intron variant	Minor allele associated with a lower incidence of pulmonary hypertension in patients with bronchopulmonary dysplasia.	[44]
DDAH2				
rs805304		2 kb upstream variant	Minor allele associated with higher prevalence of hypertension, higher ADMA concentration in diabetic renal impairment, and decreased risk of myocardial infarction.	[25,26,28]
rs2272592		2 kb upstream variant	Minor allele associated with type 2 diabetes.	[27]
AGXT2				
rs37369	Exon 4	Missense variant	Minor allele associated with higher methylarginine levels, higher diastolic blood pressure, and higher risk of coronary artery disease.	[21,33,45]
rs16899974	Exon 14	Missense variant	Minor allele associated with higher levels of SDMA, with atrial fibrillation and ischemic stroke, and higher risk of coronary artery disease.	[33,45,46]
PRMT1				
rs10415880	Intron 8	Intron variant	Minor allele associated with arteriovenous fistula malfunction in male hemodialysis patients.	[47]
rs975484		Promoter variant	Minor allele associated with reduced immune checkpoint gene expression; no significant assocation with ADMA in patients with subarachnoid hemorrhage nor in healthy male Chilean individuals.	[30,48,49]
